# New York Academy of Medicine

**Published:** 1857-07

**Authors:** 


### ECLECTIC DEPARTMENT,

### AND SPIRIT OF THE MEDICAL PERIODICAL PRESS.

---

*New York Academy of Medicine.—Puerperal Fever.*—On recommendation of the *Section on Obstetrics*, the subject of puerperal fever was brought up for discussion by Dr. John W. Francis, chairman of the committee.

Dr. Francis said: I find myself chairman of the committee on the subject of puerperal fever, and, thus appointed, am necessarily to open the discussion on that important disease. I shall do so with brevity, as I know not what particular points the Academy purposes the investigation to be carried. This disorder is so minutely described and illustrated in books, that all who now hear me, must be familiar with its symptoms and its treatment. I have, therefore, little of novelty to announce. Its inflammatory character as a general fact; its rapid course in most instances; its great malignity under peculiar circumstances, are acknowledged truths. I will, on these accounts, as well as to save time, venture to affirm some things which, perhaps, may not be found in the pages of every author on puerperal fever. I am not willing that this disorder should be restricted in its seat, to inflammation of the peritoneal lining, thus nosologically denominating the disorder "puerperal peritonitis." The disease in many instances, involves the substance of the womb and the intestines, and even contiguous viscera. We are told the complaint often sets in several days after the parturient condition is over. I have known it to assume the most malignant character within thirty-six hours after delivery, involving the womb, intestines, and even the bladder, and to terminate fatally with most disastrous destruction at the end of the third day. Upon examiuation, the autopsy has shown the womb contracted less than one-third of what it was in its full gravid state, its parietes, one and a quarter, or one and a-half inches in thickness, the inner lining overloaded with flocculi. This condition has been brought about from a variety of causes, all leading to a depressed condition of the patients a bad habit, vitiated air, and the concurring causes which bring on the typhoid form of this fever. I am satisfied of its contagious nature, from the authority of Gordon, and from what I have seen both abroad and at home. I will not trespass by the detail of circumstances in support of this view, at this time. I hardly think a member of the Academy of five year's practice, is among us, who has not seen this truth verified in hospital practice, perhaps, too, in the occurrence of puerperal fever in its sporadic form. In this age, when contagion is by many discarded as a cause of disease even in scarlet fever, and in measles, we must expect such incredulity would reject its influence even in the Lying-in-Hospital. I will add no more at present, but wait upon the discussion, for I apprehend that it is especially on this controverted subject of the specific character and contagious quality of puerperal fever, that the arguments of members will be expended.

Prof. Joseph M. Smith read an elaborately written paper, on the *Causes and Modes of Propagation of Puerperal Fever.*

The views of medical writers are divided as to the pathology of puerperal fever; one class holding to the opinion that the disease is an idiopathic fever *sui generis;* and the other that it is essentially a local inflammation, taking the form of peritonitis, metritis, metro-peritonitis, or uterine phlebitis; the former of these opinions have lately been revived, and, with the exception of imputing to the disease a specific contagious attribute, has received the support of able contributors to obstetric pathology.

The opinion of authors is also divided in regard to the nature of the morbific agent; one class contending that it is a specific contagious principle, elaborated within the body by a specific vital process; the other, that it is a miasm or poison, formed by the chemical reäctions which take place in the exhalations, and other matters excreted or discharged from the human body and retained in close apartments.

In rejecting the opinion that puerperal fever is a disease *sui generis*, we must, also, reject the idea of its propagation by a specific contagion. That the disease is communicable, there is no doubt; but it is so through the agency of a poison generated in a mode totally different from that peculiar to small-pox and measles.

Prof. Smith then proceeded to consider the *Special Etiology* of puerperal fever, under the following heads:

1. Causes of puerperal fever in public institutions.
2. Origin of sporadic or spontaneous puerperal fever.
3. The miasm of typhus fever produces puerperal fever.
4. The miasm of puerperal fever produces typhus fever.
5. The miasm of erysipelatous patients produces puerperal fever.
6. The miasm of puerperal fever produces erysipelas.
7. The emanations from bodies, examined after death, produces puerperal fever.
8. Puerperal fever and erysipelas are generally concomitant epidemics.
9. Are puerperal fever, erysipelas, and typhus, one and the same disease?
10. Extreme susceptibility of puerperal women to the effects of idiomiasm.

The conclusion at which Prof. Smith arrived, after full consideration of the above propositions, are,

That puerperal fever sometimes arises from the noxious air generated from the foul discharges of puerperal women in crowded and ill ventilated lying-in-hospitals; sometimes from the emanations of patients laboring under typhus fever, erysipelas, and gangrenous diseases; sometimes from the emanations from the human body dissected after death; and sometimes from the absorption of putrescent matters lodged in the uterus and vagina after parturition.

It would appear, also, from this inquiry, that the miasms of typhus, erysipelas, and puerperal fever are severally capable of producing any one, or all, of these diseases; and that they may attach themselves to the persons and clothing of midwives and physicians, and thus be transported from these sources, to the chambers of lying-in-women. The more ordinary form of disease, induced by the febrific effluvia in question, is typhus and its modifications, typhoid fevers; while puerperal fever, and hospital erysipelas, are but varieties of that disease, taking their forms from the peculiar predisposing conditions of the system.

The following facts are important:

1. Let the physician take care.
2. When it breaks out in a hospital, thorough resort to disinfecting agents and ventilation, and dispersion of patients is necessary.

Prof. Clark said: I am not so well prepared this evening to speak upon this question as I hoped to have been. I have not had the time to study my own papers; the subject has occupied my thoughts for twenty years, and commands my earnest attention at this moment, for since the last meeting of the Academy, the disease has appeared at the Bellevue Hospital; two patients have already died of it and seven are now under treatment. I name this incidentally, as I may allude to it again in speaking of the treatment.

With reference to the points that have been made thus far in the discussion, I have little to say; I am obliged to concur with Drs. Francis and Smith, in regard to the communicability of puerperal fever, whether by miasmatic infection, or by contagion, is a matter of far less importance than the fact that it can be conveyed by the physician. The mode of the conveyance has excited a great deal of speculation; but the poison has never been isolated. It is not known whether it is imbibed by the cuticle; or, as Tyler Smith has said, is breathed into the lungs, and through them, entering the blood, and thence coming forth as a halitus; or, whether it is carried in the clothes or hair.

1. As to its connection with typhus. At the present time, we have puerperal fever at Bellevue; but there is not a single case of typhus fever in the whole house: either in the male or female divisions. There has been no case on the female side for two months, and none on the male side for one month; and in the whole house, there have been only five or six marked cases during the whole of the last six months.

The epidemic, of which Dr. Vaché has given an account, in 1840, perhaps the most severe that has ever visited the Institution, was not preceded, attended, or soon followed by any marked increase of typhus or typhoid fever. The same may be said of the puerperal fever in 1851 and 1852. On the other hand, the severe typhus epidemic of 1846–7–8, came and went without any unusual occurrence of puerperal fever. Dr. Reese, will, I have no doubt, bear testimony to the latter statement. It would seem, then, that at Bellevue there are no fixed relations between these diseases.

I am aware that puerperal women have been placed in beds near those who have had typhus fever, and have died of puerperal fever; but unless these cases are adduced in sufficient numbers to balance the cases on the other side, they may be but coincidences.

No relation has been established between the typhus and puerperal fevers, as far as the City Inspector's reports are concerned. We have these reports from the year 1804; but from the year 1804 to 1829 or 1830, the number of deaths from these affections was annually too small to be of much value. It is impossible, without access to the City Inspector's office, to make an exact comparison from these reports; as all the persons dying from puerperal fever, "childbed," "convulsions," and from "abortion," are all classed under one head. But as those from convulsions and abortion bear pretty nearly a fixed relation to the population, we may assume that the fever causes the principal changes in the number from year to year.

The following figures will show the number of deaths from puerperal diseases, and from typhus and typhoid fevers, from 1830 to 1853:

| | | | | | | | |
|---|---|---|---|---|---|---|---|
| In | 1830, | we had | 59 | puerperal, | and | 53 | typhus deaths. |
| " | 1831, | " | 36 | " | " | 54 | " |
| " | 1832, | " | 60 | " | " | 84 | " |
| " | 1833, | " | 39 | " | " | 62 | " |
| " | 1834, | " | 61 | " | " | 106 | " |
| " | 1835, | " | 61 | " | " | 47 | " |
| " | 1836, | " | 76 | " | " | 117 | " |
| " | 1837, | " | 64 | " | " | 338 | " |
| " | 1838, | " | 40 | " | " | 104 | " |
| " | 1839, | " | 29 | " | " | 97 | " |
| " | 1840, | " | 66 | " | " | 144 | " |

And so on, till we come to the year 1847, which must be regarded as the *test* year.

| | | | | | | | |
|---|---|---|---|---|---|---|---|
| In | 1847, | we had | 94 | puerperal, | and | 1396 | typhus deaths. |
| " | 1848, | " | 147 | " | " | 948 | " |
| " | 1849, | " | 138 | " | " | 602 | " |
| " | 1850, | " | 152 | " | " | 472 | " |
| " | 1851, | " | 166 | " | " | 1104 | " |
| " | 1852, | " | 155 | " | " | 758 | " |
| " | 1853, | " | 147 | " | " | 541 | " |

The numbers will sometimes be found to increase together; but the proportions in which they increase and diminish, show very few analogies between the two diseases.

Prof. Clark here drew a contrast between the symptoms, duration, and lesions of the two affections, showing the wide differences which exists between them.

The relation of puerperal fever to erysipelas, at Bellevue, has long been remarked. My impression is that there *is a relation* between the two; this is the prevailing opinion there. A few days ago, when the puerperal fever broke out there, it was ascertained that erysipelas was prevailing to some extent in the surgical wards.

In looking over the City Inspector's report of deaths for fifty years, it is found that from 1804 to 1830, the ratio of mortality from these diseases is very variable; the numbers are, however, too small during this period to be of much value; but for the last twenty years, when the number from each is considerably increased, it will be remarked that they show a decided tendency to increase together, and to diminish together, the ratio of mortality varying bnt little.

*Hospital Gangrene* differs from erysipelas, probably more in its appearance and results than in its nature. It is, perhaps, erysipelas intensified, and somewhat modified in the tendency to spread over the body. But as the two occur under the same circumstances, and as they certainly have close alliances, what has been already said of one can hardly fail to be true of the other.

*Pathology.*—I am compelled to withhold my assent to the doctrine that this is a *fever*, and a fever only, under any circumstances. I have not yet seen a single puerperal woman die of an acute febrile disease of short duration, such as is usually called puerperal fever, in whom there could not be found, on post-mortem examination, some lesion of an *inflammatory* character. Dr. Gooch has expressed the opinion that this *fever* may occur *without lesions.* He says: "The experience of the last few years has taught us about peritoneal fevers (he prefers to call them peritoneal rather than puerperal) that they occur in their most malignant form and leave few or no vestiges in the peritoneum after death" Dr. Simpson has expressed the opinion that the disease occurs occasionally without lesions. "Occasionally," he says, "though very rarely, no inflammatory lesion whatever can be found in bodies of patients who have died of puerperal fever. Dr. Locock has observed several cases of this kind, and in the practice of the late Dr. Beilby I saw one very marked and rapidly fatal case of puerperal fever in which my colleague Prof. Bennett was unable to detect anywhere in the abdomen or uterus, its appendages or vessels, any trace of inflammatory action or effusion." Tyler Smith regards it as, in some cases, an *inflammation*, and in others a fever. I would say that puerperal fever has *four principal* lesions, and many of a secondary character.

1. Inflammation of the peritoneum.
2. Inflammation of veins of the body of the uterus.
3. Inflammation of lymphatics of uterus, veins not involved.
4. Inflammation of inner surface of the uterus, or endo-metritis.

I believe in every case where a full examination is made—I think in every case—one of these lesione will be found. In all cases where I have had an opportunity of examining, one has been found.

The first variety is the most common. It is found in the different stages of congestion, effusion of lymph, or the effusion of pus. One of these conditions is found in the great majority of the cases which I have examined.

2. The number of the cases in which *inflammation of the veins* exists is small. I have met with it in a few cases. Cruveilhier, in the Hospital Maternité, met with it in very few cases; seven or eight in the two years and a half. I have here the report of two cases which illustrate this form of the disease. The general character of this lesion will be better understood by the examination of the plates. (From Cruveilhier's *Pathological Anatomy.*)

3. *Inflammation of the Lymphatics.*—This is more common than inflammation of the veins. It is very often seen, to a limited extent, often met with in this limited extent when the chief lesion is peritonitis. This fact was first pointed out to me by Dr. Markoe, of this city, some years ago. At that time, however, it was supposed that it was the veins of this part that were the seat of the inflammation, but, on careful examination, it appears to be in the *lymphatics*, and not in the veins.

If Cruveilhier can be trusted (and I have met with confirmation of his opinion), when pus is met with, and can be traced to the lymphatics, the pus does not enter the circulation so as to destroy life, the lymphatic glands seem to filter it out, and prevent its transmission into the circulating blood. At least, pus can be traced in these cases into the lymphatic glands, but if the gland itself is not disorganized by the purulent deposit in its structure, it can not often be found in the tubes going off from the glands.

4. The next form of disease is that which Gooch, Simpson, and Tyler Smith regard as independent of anatomical lesion. The view which I wish to present of that class of cases is that they are probably all, in reality, pyæmia resulting from inflammation of the inner surface of the uterus. In order that what I have to say on this point may be better understood, we will consider one or two points in the anatomy of the uterus after parturition. I have here the uterus of a woman who died of pleurisy, ten days after delivery, in the practice of Dr. Grimsom. I have not thought proper to bring into this room, where there are so many practicing accoucheurs, a fresh specimen of the disease in question, though I could have done so. In 1840, it seemed to me I could demonstrate that there was no lining membrane to the uterus, but that the uterine sinuses opened on the free surface of the uterus. These open mouths can be seen in a good light in this specimen, entirely uncovered by any membrane. I can pass this instrument (a probe) into one of these open mouths, and out of the cut surface of the uterus, and if I was disposed to make a patient dissection, I could trace them into the veins going into the general circulation.

These sinuses remain open, or rather openable, for at least ten days. Why do they not bleed? I do not know as any one has made an exposition of it. If I see it correctly, these sinuses are guarded by a valvular opening like that of the ileo-cœcal valve. There are two folds, the longest is the innermost one; through this innerfold there is a muscular fibre well marked, and, also, a smaller muscular fibre running through the shorter lip; these muscular fibres are continuous with the muscular fibre of the body of the uterus; when the muscular fibre of the uterus is well contracted, the shorter lip is drawn down on the inner one so that no blood escapes. When the muscular tissue is relaxed, the mouths open in this way (illustrated by two pieces of paper, the one overlapping the other, like the two parts of the ileo-cœcal valve, and the ends approximated) thus it is that bleeding occurs when the uterus is relaxed, and hence the necessity of having the uterus contract to prevent hæmorrhage.

If the open body of one of these sinuses be lifted, a sort of sac will be found one-sixteenth of an inch or so in depth; and, looking toward the wall of the organ, another little mouth, sometimes two or more; whether that has a muscular fibre I am unable to say.

Now, sir, the point I make, is this:— Inflammation of the inner surface of the uterus is, also, inflammation of the valvular mouths of the uterine sinuses, since they are really a part of this inner surface; hence endo-metritis, as it seems to me well to denominate it, is of necessity a limited phlebitis, and inflammation of this inner surface of the uterus is of common occurrence, in one or other of its forms. Cruveilhier has described it as attended by an exudation which is soft, vascular, and areolar, the meshes of which are filled with blood clots. I have seen the exact copy of what he represents in Plate VI, of Book 4; and can state that the singular appearances there illustrated are due to fibrin, pus, and blood; the first forming a velvety surface on the interior of the uterus which holds in its fibres the other two, and that there is no proper vascularity in this exudation; it is nothing more than one of the least frequent of the results of the endo-metritis. The more common results of the inflammation, are exudations of a creamy consistency varying in color from a pale ink, through a brick dust to a brownish red, or sanious hue, with or without a firmer fibrinous layer, in contact with the uterine surface; in rare instances the color is the dark green, represented by Cruveilheir.

When this endo-metritis exists with the symptoms of puerperal fever, though peritonitis be absent, and though pus be not found in the lymphatics or veins of the uterus, yet we are not at liberty to infer that the disease is a fever and nothing else. Here is a source of purulent contamination. That pus is not found in the veins is not proof that it does not exist in the circulation; formed within the mouths of these sinuses, it would be readily washed into the circulating blood and produce the symptoms of pyæmia. Indeed, the proof of such contamination I have seen in the deposit of pus within the tissue of the liver.

I do not mean to say these four lesions are independent of one another. I do not mean to say that where there is found lymph effused, where there is peritonitis, or where there is inflammation of the lymphatics, there is no endo-metritis; for this endo-metritis is found in almost all cases; it is very rare for endo-metritis to occur without other lesions; again, it is not common for the neck of the uterus to have the same change that is found in the body, for the neck will often be found healthy, while the body is changed; this shows that the neck is subsidiary, and does not perform the same functions as the body, or, at least, is of different structure. I have here a summary of all the additional lesions I have noticed as far as I am able to judge

1. In most cases the fallopian tubes are found more or less filled with pus. 2. It is very common for serous membranes to be involved; the pleura is rarely free from hyperæmia; the pericardium is often injected; the stomach is almost always thickened and softened if not of a red color; the intestines are more or less hyperæmic, and hence the diarrhœa, which is more common than in other forms of peritonitis; the spleen is not commonly changed except where there is some complication; the kidneys are not involved in the diseased action, at least they do not give any evidence of being involved; the brain is almost always implicated, there being fluid in the cavities, and the vessels of the brain bleed freely when cut. My own researches do not enable me to demonstrate any very striking changes in the blood, it is coagulated in the heart and larger vessels.

The larger veins are often stained by the coloring matter of the blood after death; in one case, the autopsy was made one hour and a half after death, in order to see whether this staining was before or after death, no stains were observed; where the autopsies have been made within six hours after death there have been no stains. We can not demonstrate these changes in the blood which nature exhibits. In this respect, pathology is far behind nature. The graaffian vesicles are often of a muddy color, as if stained with pus; the ovaries are frequently inflated with serum, sometimes with pus. The primary lesions are in the organs of generation; the secondary are in the blood, and are found, indeed, in almost every organ of the body.

Dr. Reese remarked that a law should be enacted to prevent persons from attending on puerperal women, after attending cases of erysipelas, hospital gangrene, or autopsies. He had never seen a case which justified the view of this disease being contagious. He had not found local lesions sufficient to account for its fatal results; had twenty-nine cases in the month of March, while in charge of Bellevue; and, in the first five, puerperal fever came on in one to four hours after confinement. He gave the wards up to an assistant, who had five cases in two days and two nights; this satisfied Dr. Reese that he had not communicated the disease, and he again resumed the charge of the wards; he lost five out of twenty-nine cases. Phlebitis and toxemia occurred in two; he believes the disease to be epidemic. We have had illustrations here; when it prevailed along the east and west sides of the city, while in the northern part it was scarcely heard of.

Prof. Smith said he would like to make an enquiry of Prof. Clark, as he did not himself enter into the pathology of the question, except to say, that the disease is a fever, and not a phlegmasia, and that the lesions which are found are but complications. Prof. Clark does not discriminate between typhus in males and typhus in females. If he had excluded the typhus in males, perhaps the results would have been more equal. This exclusion of the males would alter the result.

These statistics are liable to lead us astray, unless we exclude the circumstances that modify the disease. I did not attempt to identify the diseases, typhus, erysipelas, puerperal fever, etc. I put it in the form of a query, in attempting to explain why typhus occurred, and puerperal fever did not. The legitimate and ordinary sorm of fever is typhus; where there is nothing to produce a modification, typhus and typhoid are similar idiopathic fevers, connected as one and the same disease, but meteoration at certain seasons, especially affects female constitutions, and under this influence another form of disease is produced.

Rokitansky regards typhus and puerperal fever, as incompatible. But there was one casc in Paris, where the intestinal lesions were demonstrated in a case of puerperal fever.

I have adopted the view, that this is a fever, and the local lesions are but complications. According to Prof. Clark, however, there is no case in which some lesion does not occur. Prof. Ossiander was the first to notice cases without lesion. Dr. Locock says, such cases are rare, yet they do occur. Dr. Bennett explained a case minutely, in which not the slightest lesion was found. Admitting endo-metritis to exist in all cases, it does not, to my mind, prove that this is not an idiopathic fever. Are the lesions sufficient to destroy life as rapidly as we see it? Some secret poison must be at work in the blood, poisoning the patient. When Dr. Reese related the case of Dr. Forrester attending his patients, I was struck with its analogy to the cases where several physicians attended a case of erysipelas, and afterwards attended puerperal women, who suffered from the disease.

Prof. Clark.—I will cheerfully answer Prof. Smith's inquiries. As to typhus occurring with puerperal fever in females, I am not able to give any additional facts. We know that typhus fever takes its full proportion of females,* and in epidemics more females are lost, because females are more about the sick. I know of nothing in the history of New York which would take epidemics out of this rule. In the severe epidemic of 1847, the number of deaths among females fell somewhat short of that among males. I can, if necessary, obtain the statistics on this point, but I believe the facts will be found substantially as I state them. I am glad Prof. Smith has given me an opportunity to explain a little more fully the purport of some of my remarks. I suppose I was understood to say that endo-metritis has been observed by every one, though the name is, perhaps, here used for the first time. What I intended to say is this, that I have seen, I believe, the cases which Gooch, Simpson, and others describe as puerperal (or peritoneal) fever, without lesions, and have never failed to find the evidences of endo-metritis. I did not intend to express the idea that this limited amount of inflammatory action could, of itself, by the force of its local irritation, produce this terrible fever, and destroy life so rapidly, but that this inflammation is a purulent contamination of the blood, in manner already explained, and that the patient dies, not of endo-metritis, but of pyæmia. I have here a case reported at great length, in which death occurred after six days of illness. There was no peritonitis, no inflammation of the lymphatics or veins in the substance of the uterus; but there was endo-metritis, attended by a gruel-like exudation, of brick-dust color, which contained pus, fibrin and blood. The tissue of the uterus, to a very minute depth from within outward, was stained of a reddish brown color. But the most important fact was that the liver presented numerous yellowish points, which, on microscopic examination, were found to contain pus, and in the centre of each one of these points was a capillary vessel; this was, in all probability, a secondary deposit, and the endo-metritis was the spark which kindled the fire. Thus I am led to sappose it must be in other cases, where this lesion has been overlooked. Not that there are secondary deposits in each case to testify to the violence of the disease—for these are rare—but there is a true pyæmia. A case of otorrhœa, with infiltration of pus into the jugular vein, and death in five days was adduced. Many of these cases are rapidly fatal, others are not. I do not wish to be understood as excluding from this disease a febrile element, for that it unquestionably has; but, so far as my observation goes, it is accompanied by, if not dependent upon, a local lesion. I saw the erysipelas that prevailed throughout the country ten years ago; it began with a febrile movement. The febrile movement was a part of the disease, and preceded it. There is one point that is worth alluding to, viz: the absorption of the decomposed element in the uterus; this may be so, but I suppose the great cause of death is pyæmia.

Dr. Cock inquired of Prof. Clark if his investigations had been carried any farther than the appearances after death. Has he made any investigations, in the ordinary discharges of the lochia, whether they do not contain pus? Ferguson, many years ago, called these secretions pus, and he believed the veins of the uterus were the sources of the poison. Puerperal fever manifests itself, also, in a minor manner in phlegmasia dolens. I saw, with one of my Bellevue friends, one of these cases; she was then unequivo-

*See Bartlett *on Fevers*, 4*th* Ed., pp. 126 and 253.

cally in puerperal fever from phlegmasia dolens; it was a few hours before death.

Prof. Clark replied that he had appreciated the importance of the fact referred to in Dr. Cock's first question—had used such opportunities as he had met with to ascertain whether pus was produced in the uterus in other fatal puerperal diseases. In three or four cases of convulsions he had not found it. Second, the contamination from purulent inflammation of the veins is not a case of the class falling under puerperal fever; it is allied to phlebetis in other parts of the body. A married lady who, it was thought, was threatened with convulsions, was bled; red lines followed from the point of venesection; she coughed, and had blaody expectoration, and, if she had lived long enough, would have expectorated pus, from the formation of metastic abscesses in the lungs. Those cases are therefore excluded.

(To be continued.)

